# Overview of Contemporary Penile Rehabilitation Therapies

**DOI:** 10.1155/2008/481218

**Published:** 2008-09-04

**Authors:** Peter Hinh, Run Wang

**Affiliations:** Division of Urology, The University of Texas Health Science Center at Houston, 6431 Fannin Street, Houston, TX 77030, USA

## Abstract

*Introduction*. Post-prostatectomy erectile dysfunction affects a considerable number of men and is a significant quality of life issue. There has been a substantial amount of research on the treatment of post-prostatectomy ED, and now there is a rising interest in the concept of penile rehabilitation. The goal of penile rehabilitation is to moderate the destructive processes that occur after prostatectomy in order to preserve erectile function, either through spontaneous or assisted means. *Methods*. We reviewed published data and experiences of post-prostatectomy penile rehabilitation using regimented interventions of phosphodiesterase inhibitors, vacuum erectile device, and intracavernosal agents, and we present and analyze the research conducted. *Results*. These studies show improved objective and subjective clinical outcomes in regards to physical parameters, sexual satisfaction, and rates of spontaneous erections. *Conclusion*. These studies are often limited by small size, study period, and study design. There continues to be a need for large, randomized, placebo controlled trials with adequate followup to fully evaluate the efficacy and cost-effectiveness of the various proposed penile rehabilitation regiments before a clear standard can be established.

## 1. INTRODUCTION

Erectile dysfunction following prostatectomy remains a significant quality of life issue for men undergoing prostatectomy. It is estimated to affect 26–100% of patients after surgery [1]. Even with advancements in understanding the anatomy of the prostate and the neurovascular bundle [2], a considerable number of men undergoing prostatectomy will have resulting erectile dysfunction.

Progress has been made in identifying the events that contribute to erectile dysfunction after prostatectomy. The changes of neuropraxia, ischemic and hypoxic insults, fibrotic remodeling, and apoptosis are all believed to contribute to erectile dysfunction [3, 4]. These events can occur even in attempts at meticulous dissection to preserve the neurovascular bundle. The etiologies of cavernous nerve neuropraxia include mechanical stretch injury during retraction, ischemia from accessory vessel disruption in dissection, thermal injury from electrocautery use, and inflammation from surgical trauma. This neuropraxia can prevent erections, and the perpetual lack of erection can itself set up a cascade of deleterious processes. Chronic impotence reduces blood flow to the corporeal bodies, which leads to fibrosis and transformation of the trabecular smooth muscle through collagen [5]. Further hypoxic insults also may trigger apoptosis [6]. Therefore, the goal of penile rehabilitation is to set up an environment that moderates these processes in attempt of preserving penile function and earlier return of potency. Regimented usage of erectile aids aims to improve the circulation of oxygen and maintain the structure of the corporeal bodies.

However, the research has yet to be translated into a coherent clinical strategy for penile rehabilitation. As such, there are no currently accepted guidelines for penile rehabilitation regiments. Certainly, there exist several popular options that are currently in use. In practice, three principle modalities of treating post-prostatectomy erectile dysfunction are employed. This paper will review the efficacy of these options in this patient population for the purposes of penile rehabilitation.

## 2. PHOSPHODIESTERASE 5 INHIBITORS

The introduction of phosphodiesterase inhibitors (PDEi) revolutionized the treatment of erectile dysfunction. Since entering the market in 1998, these medications have become nearly synonymous with erectile dysfunction. Their ease of use and relatively safe profile have made them pervasive in the treatment of erectile dysfunction. They have also been extensively investigated. A Cochrane meta-analysis looking at many large, randomized clinical trials concluded that PDEi are efficacious in the treatment of erectile dysfunction and are generally safe [7]. However, their role and their administration in penile rehabilitation after prostatectomy remain undefined. A number of clinical studies have investigated PDEi use in this population for this intention.

One of the first studies on PDEi in rehabilitation looked at objective data to support this use. Schwartz et al. conducted a study on 40 men who had undergone nerve sparing prostatectomy [8]. Prior to prostatectomy, all men had percutaneous biopsy of cavernous tissue to serve as baseline reference. They were divided into receiving either 50 or 100 mg of Sildenafil every other night. Participant then underwent percutaneous biopsy of cavernous tissue at 6 months to compare with baseline tissue. Investigators found that the 50 mg group did not experience any loss of smooth muscle compared with baseline, and the 100 mg group actually showed an increase of smooth muscle content when compared to the baseline. There was no control group, and no clinical correlation between smooth muscle preservation and erectile function is made in this study. A prior animal study had shown that cavernosal smooth muscle does atrophy after prostatatectomy [9], though this effect is somewhat mediated with unilateral nerve sparing and it is unclear to what extent this atrophy would occur with nerve sparing prostatectomy.

Bannowski et al. conducted a randomized trial following 43 men who underwent nerve-sparing radical prostatectomy [10]. All men provided baseline International Index of Erectile Function (IIEF) scores prior to surgery. After catheter removal following surgery, the men underwent testing for nocturnal tumescence the following evening measured by the rigiscan. 41 of 43 were found to have spontaneous erections on rigiscan, and these men were then randomized to receive sildenafil daily or no treatment. They were then followed and evaluated with IIEF at 6, 12, 24, 36, and 52 weeks. The results show that the daily treatment group had significantly higher IIEF score by 36 and 52 weeks. Additionally, 47% of the daily treatment groups were able to achieve spontaneous, unassisted erection sufficient for penetration. This compares to 28% of the control group who were able to have such erections. Both groups were also allowed Sildenafil on demand, and accounting assisted erections, 86% of the daily group had erections sufficient for penetration, compared to 66% of the control group. The study made no mention of any participant drop out, and there was no placebo control. However, it does appear that daily Sildenafil does improve return of spontaneous erections and can augment response to on-demand use of Sildenafil.

A stringent, randomized, double-blinded, placebo controlled study was performed by McCullough et al. evaluating the efficacy of daily Sildenafil in men after bilateral nerve-sparing radical prostatectomy [11]. This study included 54 men with baseline normal EF and NPTR (nocturnal penile tumescence and rigidity with duration of rigidity >55% of maximal rigidity using penile plethysmography). After a pretreatment period of 4 weeks, they were then randomized to either receive nightly 100 mg Sildenafil (*N* = 18), 50 mg Sildenafil (*N* = 17), or placebo (*N* = 19). The groups were then analyzed at 16, 28, and 40 weeks. Then after 40 weeks, all medications were discontinued and the groups were again analyzed at 48 weeks. At each point, the participants were evaluated by NPTR and IIEF . The study found that the groups receiving daily Sildenafil were able to have return of rigidity (*R* > 55%) at seven times the nadir value compared to minimal improvement in the control group. This improvement of erection was also seen by the investigators for RAU (rigidity-activated unit—a time-intensity measurement that represents the area under the rigidity curve during a qualified event), with the additional finding that the 100 mg group experienced continued improvement after the discontinuation phase, while the 50 mg group began to experience decline in RAU. Importantly, the men on daily Sildenafil were five times more likely to have return of spontaneous, unassisted erection sufficient for intercourse compared to placebo. The authors were able to link objective measurements of erections with subjective, clinical response. They noted that tip rigidity >55% clearly separated responders versus nonresponders (responders defined as recovery of spontaneous, unassisted sufficient erections). This study may mark objective validation as an important component of future clinical trials. These initial results are from a subset analysis performed on men who showed normal EF and NPTR on baseline, and we await further data and analysis on the entire study patients.

Taken together, these studies seem to indicate that phosphodiesterase inhibitors have a role in penile rehabilitation for men after prostatectomy. There may also be a dose-dependent relationship between the medication and outcomes. The studies also confirm the tolerability and safety of such a regiment, as discontinuation rates were very minimal and no adverse events were reported.

## 3. VACUUM ERECTILE DEVICE

The Vacuum erectile device assists erections by drawing blood flow into the cavernous sinuses through negative pressure, physically causing an erection. A constrictive band can also be placed at the base of the penis, preventing backflow and maintaining corporal pressures. This direct mechanism of action can circumvent the limitation of oral agents, which requires an intact and functioning neuronal connection to produce erections. This can be a significant factor even in men undergoing nerve sparing prostatatectomy, as neuropraxia still occurs and can diminish the effectiveness of PDEi.

This treatment modality can also be extended to men who have undergone nonnerve sparing prostatectomy, though not in the context, in penile rehabilitation with the expectation of return of potency.

If not for potency itself, VED usage has also been advocated due to its possible efficacy in preventing penile shrinkage and maintaining length. Studies have shown significant shrinkage of penile length, with one study finding that nearly 20% of men experience a loss of length greater than 15% [12]. In another study examining penile shortening after prostatectomy, Gontero et al. followed 126 men who had undergone prostatectomies and measured penile length prior to surgery, at the time of catheter removal, and then at 3, 6, and 12 months [13]. They found that the greatest amount of shrinkage occurs in the immediate postoperative period, though shortening continues at a lesser rate throughout the entire study period. These authors hypothesize that early hypoxia leads to increased expression of TGF-B and Collagen I and III fibers. This study also finds that the return of erectile function, defined as an IIEF of 15, was associated with mitigation of the shrinkage, as well as having a nerve sparing surgery. Several studies looking at the efficacy of vacuum erectile device in preserving erectile function have also examined preserved penile length as a secondary endpoint.

Raina et al. randomized 109 post-prostatectomy men to either early VED use daily (*N* = 74) versus no erectogenic aid (*N* = 35) [14]. The men were to use the constriction band only during intercourse to maintain rigidity. Participants were followed with SHIM and IIEF scores for comparison. For the group using VED, 80% were able to achieve penetration with use of VED, and this group, not surprisingly, had a significantly higher SHIM and IIEF group compared to no treatment. The discontinuation rate was 18%, and the majority of the drop out was for discomfort. In the context of penile rehabilitation, at 9 months this study found that 17% of those adhering to daily VED were able to have spontaneous erections sufficient for erections at 9 months, compared to 11% (*n* = 4) of the control group that had such erections. In regard of the effect of VED on penile length, the men who adhered to VED regiment experienced less subjective penile shrinkage, with 23% reporting less length compared to 85% of the men who quit treatment and 65% of the control. No objective data was collected concerning length. The authors conclude that early use of VED with the purpose of penile rehabilitation improves sexual and partner satisfaction and allow for earlier return of spontaneous erections. Although, the rate of return of spontaneous erection is low for both groups, those numbers include men who had undergone nonnerve sparing prostatectomies. This distinction is necessary, as penile rehabilitation is more directed for NS men and the inclusion of nonnerve sparing prostatectomy patients diluted the response to rehabilitation.

The timing of when to initiate VED has been questioned, with some advocating an earlier intervention. Köhler et al. randomized 28 men to either receive early VED regiment (1 month after RP) or delayed VED regiment (6 months) [15]. The regiment consisted of 10 minutes of VED usage without the constriction band. IIEF was measured at baseline, 1, 3, 6, 9, and 12 months. The mean followup was 9.5 months and the results analyzed at 3 and 6 months showed that the early intervention group had a statistically higher IIEF score. At this point, the comparison shows that VED does improve IIEF scores among those who use VED versus those controls that do not. At beyond 6 months, the delayed group began using of VED, and at short followed up the two groups converged with no statistical difference in IIEF categorization. No patients in this study had return of spontaneous erections sufficient for penetration at that followup. This paper was presented as a pilot study, and more outcomes are expected to follow, especially data concerning return of spontaneous erections. There are still some important points that can be gleamed from this study. For one, the authors reported complete compliance with this regiment, suggesting this short regiment (two five-minute cycles) could be feasibly implemented. Importantly, these researchers also looked at penile length and found that the group performing VED regiment did not experience penile shrinkage, while the group on delayed VED showed significant penile shrinkage at 3 (mean loss 1.87 cm) and 6 months (mean 1.82 cm). However, after beginning VED in the delayed group, the loss decreased to a mean of 1 cm and no longer remained statistically significant. Again, this is short-term followup data in the delayed group, and further improvement in shrinkage may still yet be seen. We still await data concerning return of spontaneous erection from this study.

The value of VED in penile rehabilitation remains uncertain. Daily regimented use of VED requires a motivated patient and does improve sexual satisfaction in those who responds. If the stated goal of penile rehabilitation is the return of preexisting potency, then further studies are needed to show that VED improves the rate of return of erection. However, VED use may also be advocated for its effects on preventing penile shrinkage after prostatectomy.

## 4. INTRACORPOREAL INJECTION

Intracorporeal injection of vasoactive agents increase blood flow into the cavernous sinuses locally, either through increasing cAMP, by antagonizing alpha-adrenergic receptors, or by direct smooth muscle relaxation. Like VED, they also do not require an intact, functional nervous system to produce erections. Thus, they can also be offered in men who have undergone nonnerve sparing surgery and men who do not respond to oral agents.

Montorsi et al. conducted a randomized trial investigating whether a regiment of intracavernosal injections improves erectile function in post-prostatectomy men [16]. 30 men with established preoperative potency were randomized to either receive a regiment of 3 times per week injections of alprostadil for 12 weeks versus a control group that did not receive erectogenic treatment. The groups were then assessed after 3 months for sexual history, for Doppler response after alprostadil administration, and for nocturnal tumescence. Of the ICI regiment group, 80% completed the 12 weeks of treatment with a 20% drop-out rate and a 17% complication rate. Of these men, 67% at 3 months were able to have spontaneous erections sufficient for penetration. This favorably compares to 20% of the control group men that were able to have such erections. The authors do note that in this group some still continued to use ICI to achieve erections. However, the authors considered it a complete response since the majority of sexual encounters occurred without ICI use (average of one in 4.2 attempts). The study also found that having normal penile hemodynamics was strongly associated with ICI complete response. The study suffers from small size and short followup. However, it was still able to show an improvement in spontaneous erections for regimented ICI and that regimented ICI is generally well tolerated.

Mulhall et al. conducted a trial that may be more clinically applicable, even though it was not randomized [17]. In this trial, post-prostatectomy men were committed to penile rehabilitation with Sildenafil or ICI if there was no response to Sildenafil, versus no penile rehabilitation program, but they were not restricted from using erectile aids. The rehabilitation group used either Sildenafil or ICI three times per week. Analysis at 18 months revealed that 52% of rehabilitation men were able to have functional erections, compared to 19% of control group men. Additionally, the rehabilitation group had significantly higher IIEF scores. These results are impressive, especially considering that the control group was also able to use erectile aids, including both Sildenafil and ICI, though not in a regimented manner. The study included men with nonnerve sparing prostatectomies, which generally are not considered candidates for erectile rehabilitation, though the authors do note that there was not a differential distribution between the groups. Additionally, the average length of time before sexual consultation and therefore the start of rehabilitation were 4.2 months. Some would suggest that this length of time is too long removed from the surgery, and the insults of hypoxia, fibrosis, and apoptosis may have already occurred and be irreversible at this point. It is also important to note that selection bias may be very considerable in this study, in that only men who prospectively committed themselves were included in the rehabilitation group and the study relied on mainly self-reported, subjective data.

## 5. COMBINATION THERAPIES

Studies have examined the feasibility and efficacy of employing two treatment modalities during penile rehabilitation. Nandipati et al. incorporated both intracavernosal therapy and PDEi in a group of 22 men who underwent nerve sparing prostatectomy [18]. All participants received Sildenafil 50 mg daily (25 mg if subjects complained of headaches). For ICI, 18 patients received PGE 1–4 micrograms and 4 received trimix injection, with ICI being done two to three times per week. These patients were then analyzed at 3, 6, 9, and 12 months with IIEF . Doppler studies were performed for dose optimizations of ICI and at intervals to increase dosage for response. The study reported a mean followup of six months. At this point, investigators found that 21 of 22 patients were sexually active, while 12 of the 21 were using ICI alone and 9 of 21 were using combination therapy. Of the 18 patients using PGE, 12 were able to lower their dosage; while 1 of the 4 patients on trimix was able to do so. 11 of the 22 men had return of spontaneous erection, though none graded the erections sufficient for penetration. The authors concluded that the addition of Sildenafil could reduce the amount of ICI necessary to achieve erections. This study had a lower rate of return of functional erections compared to other studies looking at nightly PDEi 1 [11] or regimented ICI alone [16], and this difference may be explained by the shorter followup and the small number of participants. Without proper study design, in such combination therapy studies, it is difficult to assign particular findings to specific intervention.

## 6. NOVEL THERAPIES

Other therapies outside of these three mainstream modalities have been investigated for penile rehabilitation after prostatectomy. Recently, investigators in Korea looked at statins for treating erectile dysfunction after prostatectomy [19]. The basis for this hypothesis stems from the known protective effect on vascular endothelium and increased NO activity. The researchers randomized 50 men post-prostatectomy to receive 10 mg of atorvastatin for 90 days. All men were then to use Sildenafil 50 mg per day on demand. The men all had superior function prior to the surgery with IIEF 25. At 6 months of followup, the study found that the statin group had more patients categorized at potent ( IIEF greater than 16) with 11 in the statin group and 6 in the control group. Additionally, more men in the statin group were able to achieve vaginal penetration without PDEi than in the control group (8 versus 4), though this significance did not reach statistical significance. The study was neither blinded nor placebo-controlled. It is important to note that the inclusion criteria were extremely stringent, and patients with significant cormidities were excluded. This may affect the applicability or generalizability of the results.

## 7. CONCLUSION

Sexual potency after prostatectomy remains a significant quality of life issue after prostatectomy. There exist many studies on the efficacy of the various treatment options on this patient population. There are much less data looking on the effects of regimented usage of PDEi, VED, and/or ICI in improving erectile function in this group, and no guidelines exist to help steer the clinician. We are still in need of large, randomized, controlled, clinical trial with adequate, long-term followup to evaluate this question. Moreover, even after each treatment can be established to be efficacious in penile rehabilitation, the exact regiment amount and duration will still be open to further investigations for optimization. This is a especially important question in dealing with phosphodiesterase inhibitors, where the cost of treatment is substantial. However, even though currently sparse, the consistent, growing body of evidence does support penile rehabilitation in improving return of sexual functioning. As awareness of penile rehabilitation increases and becomes more accepted, it is becoming more difficult to conduct placebo or nonintervention controlled trial. There are still several ongoing trials evaluating penile rehabilitation, including a large multicenter study examining penile rehabilitation with medicated urethral system for erection (MUSE), and several of the studies presented here will further analyze with additional data from which the urology community may further define when and how to implement penile rehabilitation in post-prostatectomy men.

Currently, the body of evidence does seem to suggest a beneficial role for penile rehabilitation after prostatectomy in improving return of potency. Such a program should begin with a detailed evaluation on the preoperative sexual performance characteristic of the patient and then a thorough discussion of the available rehabilitation regiments. The practitioner should consider factors that are important to the patient including ease of use and compliance, patient motivation, conditioning, cost and patient expectations about sexual function, and penile length. Penile rehabilitation may continue to remain investigative until more standardized clinical data becomes available.

## Figures and Tables

**Table 1:** Summary table of penile rehabilitation trials.

Authors	Year published	Treatment regiment	Study design	*N*	Significant findings
Schwartz et al.	2004	QOD PDEi	Prospective	21	No loss of smooth muscle in 50 mg group, gain of smooth muscle in 100 mg group

Bannowski et al.	2008	Daily PDEi	Prospective, randomized control	41	Treatment group had significantly higher IIEF and higher spontaneous erection rates

McCullough et al.	2008	Daily PDEi	Prospective, randomized, placebo control	54	Treatment groups had higher return of rigidity, higher rate of spontaneous erections

Raina et al.	2006	Daily VED	Prospective, randomized control	109	Improved sexual satisfaction, higher rate of spontaneous erections

Köhler et al.	2007	Daily VED (10 mins), immediate versus delayed	Prospective, randomized	28	Delayed use of VED did not affect sexual satisfaction once use began. There is no statistical significance in penile shrinkage once VED started

Montorsi et al.	1999	ICI 3 times weekly	Prospective, randomized control	30	Higher percentage of treatment group having spontaneous erections

Mulhall et al.	2005	ICI or PDEi to achieve erections 3 times weekly	Prospective, control	132	Treatment groups had 2.7 times the rate of spontaneous erections, statistically higher IIEF scores

Nandipati et al.	2006	Daily PDEi and ICI 2-3 times week	Prospective	22	Assisted early sexual activity and satisfaction. Addition of PDEi allows lower dose of ICI.
